# Prof. Dr. med. Florian Eitel, 13 March 1943 – 9 April 2015

**DOI:** 10.3205/zma000967

**Published:** 2015-05-13

**Authors:** Martin R. Fischer

**Affiliations:** 1 Gesellschaft für Medizinische Ausbildung, Vorsitzender des Vorstandes, Erlangen, Deutschland; 2 Klinikum der Universität München, Institut für Didaktik und Ausbildungsforschung in der Medizin, München, Deutschland

## Goethe, Faust: The First Part of the Tragedy

“In life’s tide currents, in action’s storm,

Up and down, like a wave,

Like the wind I sweep!

Cradle and grave –

A limitless deep –

An endless weaving

To and fro,

A restless heaving

Of life and glow, –

So shape I, on Destiny’s thundering loom,

The Godhead’s living garment, eternal in bloom.”

(*translated by Charles Timothy Brooks, 1868*)

## Obituary

Far too early at the age of 72, Professor Florian Eitel passed away on April 9^th^, 2015 following a severe illness. We, the members of the *Gesellschaft für Medizinische Ausbildung*, mourn the loss of our honorary chairman. Florian Eitel was a highly dedicated surgeon, scientist and courageous pioneer in the field of medical education, who was always seeking and finding new possibilities for reforming university medical programs to improve the quality of teaching. Florian Eitel actively shaped the GMA as its chairman from 1993 to 2003 and set the course for the positive developments seen in our organization since. He also received great international recognition, particularly for his commitment to the Association for Medical Education in Europe (AMEE) and on the World Federation of Medical Education (WFME) task force for developing global standards for medical education. His efforts to make the progress in the German-speaking countries regarding improvements in medical teaching visible in the international context were not only significant, but also successful. He was active up until the end on the GMA Committee for Accreditation and Certification and critically scrutinized learning objectives and their influence on the quality of medical education in his unfinished memorandum on the National Catalogue of Competency-based Learning Objectives for Undergraduate Medical Study (NKLM). Florian Eitel was an independent and occasionally uncomfortable thinker and debater, who placed great value on the clarity of definitions and concepts, and as such encouraged many to reflect and critically respond.

To make this discourse heard, Florian Eitel rendered outstanding service to the *Zeitschrift für Medizinische Ausbildung* (ZMA). He saw to it that the journal, Medizinische Ausbildung, was published by the Thieme Verlag Stuttgart as a supplement to Das Gesundheitswesen. The first issue appeared in May 1998. He dedicated himself with vigor to the goal of having the journal listed in the international citation indexes and promoting the scientific debate within teaching and learning in the health sciences. His efforts bore fruit and the journal has successfully developed into the central journal for research in medical education in the German-speaking countries.

Florian Eitel completed his medical studies in 1969 at the Saarland University (Homburg) and earned the title Dr. med. in 1971. Following his military service as medical officer, he worked in the surgical department of the University Hospital in Homburg/Saar and became a specialist physician for surgery and senior physician there in 1975. In 1977 he expanded his expertise to include trauma surgery. In 1981 he qualified as professor and followed his head, Professor Schweiberer, to Ludwig-Maximilians-University (LMU) Hospital in Munich. Since an occupational disease increasingly hindered him in his clinical practice, he established a specialty in theoretical surgery at LMU. In 1987 he was appointed adjunct professor and founded an interdisciplinary working group on university teaching. He has since served in many ways on diverse committees and projects at the national and international levels to improve medical education.

Florian Eitel very successfully secured funding for reform projects, thus giving impetus for the development of models that have been adopted and used by others. Particularly worthy of note is his committed participation in the Murrhardter Circle at the Robert Bosch Foundation, which to this day has formulated effective reforms in *Das Arztbild der Zukunft*. An important role is played by the Munich Curricular Innovation Project (M-CIP) with its new, student-centered approaches to teaching.

In 1996 Florian Eitel wrote in Medizinische Ausbildung that hindsight explains current situations and imparts knowledge and experience that can be meaningful for the future. This remains true today.

Our deepest gratitude is due to Florian Eitel for his work and successful efforts to improve education and teaching. He will be missed as a colleague, comrade, and friend, and we will hold his memory dear in our hearts. To his family we extend our deeply felt sympathy and to him our unceasing commemoration.

